# A call to action in improving access to diabetes care in lower- and middle-income countries

**DOI:** 10.1016/j.lanwpc.2024.101187

**Published:** 2024-08-24

**Authors:** Janine Audrei T. Pajimna, Giannina Alyana L. Orpilla, Mark Jason D.C. Milan, Denise Joy Emmanuelle C. Lopez, Camille K. Pascasio

**Affiliations:** a Department of Medicine, St. Luke’s Medical Center-Quezon City, 279 E Rodriguez Sr. Ave, Quezon City, 1112 Metro Manila, Philippines; b University of Santo Tomas, España Boulevard, Sampaloc, Manila, 1008 Metro Manila, Philippines; c Department of Pediatrics, University of the Philippines-Philippine General Hospital, Taft Avenue, Ermita, Manila, 1000 Metro Manila, Philippines; d Division of Endocrinology, Diabetes and Metabolism, Department of Medicine, Philippine General Hospital, University of the Philippines-Philippine General Hospital, Taft Avenue, Ermita, Manila, 1000 Metro Manila, Philippines; e Department of Medicine, St. Luke’s Medical Center-Global City, 5th Avenue, Taguig, 1634 Metro Manila, Philippines

The recent study by Wang et al. published in *The Lancet Regional Health – Western Pacific*^1^ reported that the use of real-time continuous glucose monitoring (CGM) in hospitalised patients with diabetes receiving short-term continuous subcutaneous insulin infusion resulted in better glucose control compared to the present standard point-of-care (POC). These findings provide further support for current international consensus regarding the use of CGM in all patients with type 1 diabetes and those with type 2 diabetes mellitus on intensive insulin therapy (IIT) who are not achieving glucose targets. However, despite presence of abundant data in support of CGM, its use in lower- and middle-income countries (LMIC) such as the Philippines remains limited. Some of the challenges of CGM adoption in the country include unaffordability, high illiteracy rate, reimbursement constraints, lack of healthcare professionals promoting and implementing use of CGM, and inexperience of both patients and clinicians.^2^ The country’s national health insurance system also has very limited coverage for diabetes care. Insulin, as well as diabetes management devices and related supplies like test strips, are not currently covered.

Although CGM was associated with higher overall costs, multiple health economic analyses^3^, ^4^, ^5^ have actually found it to be a more cost-effective option compared to POC. Improvement in long-term clinical outcomes resulted in bigger savings in terms of mean per-patient lifetime costs associated with hypoglycemic events and cardiovascular, renal, and ophthalmic complications due to lower incidence of long-term complications.^4^

In line with this, measures must be taken to improve patient access to diabetes care, especially in LMICs which face substantial economic burdens due to the effects of the chronic disease. Local governments could potentially collaborate with private sectors to make CGM more accessible, and economic analyses can also spearhead the amendment of national health policies for a better reimbursement system of diabetes technology.

CGM is an educational and empowering tool that promotes proactive awareness and self-management. As diabetes technology advances, national health policies clearly lag behind. Better efforts should be made to make CGM more accessible in LMICs.

## Contributors

All authors contributed to the writing of this manuscript. All authors approved the final version of the manuscript and had final responsibility for the decision to submit for publication.

## Declaration of interests

The authors declare no competing financial interests or personal relationships that could have appeared to influence the work reported in this paper.
